# Toward Patient-Specific, Biologically Optimized Radiation Therapy Plans for the Treatment of Glioblastoma

**DOI:** 10.1371/journal.pone.0079115

**Published:** 2013-11-12

**Authors:** David Corwin, Clay Holdsworth, Russell C. Rockne, Andrew D. Trister, Maciej M. Mrugala, Jason K. Rockhill, Robert D. Stewart, Mark Phillips, Kristin R. Swanson

**Affiliations:** 1 Department of Neurological Surgery, Feinberg School of Medicine, Northwestern University, Chicago, Illinois, United States of America; 2 Department of Radiation Oncology, University of Washington Medical Center, Seattle, Washington, United States of America; 3 Department of Radiation Oncology, Brigham and Women’s Hospital, Boston, Massachusetts, United States of America; 4 Sage Bionetworks, Seattle, Washington, United States of America; 5 Department of Neurology, University of Washington Medical Center, Seattle, Washington, United States of America; 6 Department of Engineering Sciences and Applied Mathematics, McCormick School of Engineering, Northwestern University, Evanston, Illinois, United States of America; Dresden University of Technology, Germany

## Abstract

**Purpose:**

To demonstrate a method of generating patient-specific, biologically-guided radiotherapy dose plans and compare them to the standard-of-care protocol.

**Methods and Materials:**

We integrated a patient-specific biomathematical model of glioma proliferation, invasion and radiotherapy with a multiobjective evolutionary algorithm for intensity-modulated radiation therapy optimization to construct individualized, biologically-guided plans for 11 glioblastoma patients. Patient-individualized, spherically-symmetric simulations of the standard-of-care and optimized plans were compared in terms of several biological metrics.

**Results:**

The integrated model generated spatially non-uniform doses that, when compared to the standard-of-care protocol, resulted in a 67% to 93% decrease in equivalent uniform dose to normal tissue, while the therapeutic ratio, the ratio of tumor equivalent uniform dose to that of normal tissue, increased between 50% to 265%. Applying a novel metric of treatment response (Days Gained) to the patient-individualized simulation results predicted that the optimized plans would have a significant impact on delaying tumor progression, with increases from 21% to 105% for 9 of 11 patients.

**Conclusions:**

Patient-individualized simulations using the combination of a biomathematical model with an optimization algorithm for radiation therapy generated biologically-guided doses that decreased normal tissue EUD and increased therapeutic ratio with the potential to improve survival outcomes for treatment of glioblastoma.

## Introduction

Glioblastoma (GBM) is a primary brain neoplasm characterized by rapid growth and extensive invasion of the surrounding brain, with a median survival time of 14.6 months from diagnosis [1]. Upon clinical presentation, patients often undergo magnetic resonance imaging (MRI) for preliminary radiographic diagnosis. In most cases, another MRI is obtained for surgical planning, followed by a treatment regimen that includes surgery, radiotherapy and chemotherapy. The standard-of-care consists of conformal radiation delivery (35 daily fractions of 1.8 Gy for a total of 63 Gy) to the bulk tumor plus a 2–3 cm margin to include invasive disease. This treatment approach results in dose plans that are spatially uniform over the target volume and do not account for patient-specific biological heterogeneity. That is, this approach includes the irradiation of large volumes of normal brain tissue that has been invaded by an unknown but low cell density of tumor cells. Thus, depending on the patient-specific degree of diffuse invasion of these margin areas peripheral to the abnormality seen on clinical imaging, the amount of normal tissue irradiated may or may not correlate with the extent of tumor cell burden found in the margin.

Recent advances in radiotherapy planning and treatment delivery, such as intensity-modulated radiation therapy (IMRT) and stereotactic body radio therapy, have made it possible to deliver spatially non-uniform doses with very steep gradients between high and low dose regions, capable of improved normal tissue sparing. When combined with patient-specific biological information, these treatment modalities have the potential for substantially improved therapeutic ratios, i.e. the ratio of dose to tumor versus dose to normal brain [2]–[6].

In this work, we use a proliferation-invasion-radiotherapy (PIRT) model [7] of GBM growth and response to therapy to account for patient-specific tumor proliferation and invasion kinetics combined with a multi-objective evolutionary algorithm (MOEA) for IMRT optimization [8] to demonstrate the potential to improve tumor control while reducing dose to normal tissue relative to the standard-of-care. Specifically, we applied the methodology presented in Holdsworth et al (2012) [8] with a more realistic set of optimization inputs, to a cohort of 11 GBM patients, and compared patient-individualized, optimized IMRT plans with standard-of-care plans. Across this cohort of GBM patients with diverse imaging patterns, we predicted the benefit of the patient-individualized, optimized plans in terms of normal tissue dose, therapeutic ratio and simulated treatment benefit.

## Materials and Methods

### Ethics

This study, and the written, informed consents obtained from all patients were approved by the local institutional review boards at the University of Washington and the University of California Los Angeles.

### Equivalent Uniform Dose (EUD)

There are many biological metrics that can be used to quantify dose-volume effects in the brain and compare radiation doses with different spatial distributions. In this manuscript, we chose to use equivalent uniform dose (EUD) as it emphasizes changes in the overall sensitivity of the whole brain to large changes in fraction size. However, the algorithm presented discussed here can easily accommodate any metric or other criteria.

EUD is defined to be the uniform dose that yields the same cell-density-weighted surviving fraction as the non-uniform dose [9]. To compute this, we solve for the EUD such that:
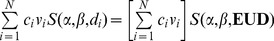
(1)where *c_i_*, *v_i_* and *d_i_* represent the cell density, volume and fraction size, respectively, at the spatial location indexed by *i* where N = 1000, the number of spatial locations in **x**.

### PIRT Model

Following [7], [10] our patient-specific mathematical model for gliomas proliferation, invasion and response to radiotherapy is formalized as a reaction-diffusion equation for the spatio-temporal evolution of tumor cell density (*c*) in terms of the net rates of proliferation (*ρ*), invasion (*D*) and radiosensitivity (*α*,β):
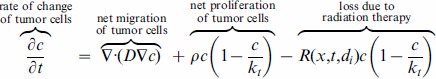
(2)where



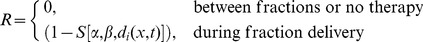
(3)In words, the rate of change of the glioma cell density *c* (cells/mm^3^) at time *t* and location *x* is determined by the net dispersal of glioma cells at a rate *D* (mm^2^/year) and net proliferation of glioma cells at a maximum rate of *ρ* (year^−1^). The net proliferation is saturated when the local tumor cell density reaches the carrying capacity *k_t_* where
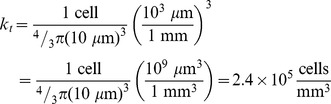
(4)is the maximum theoretical density assuming a tumor cell radius of 10 µm [11]. The radiation loss term *R(* ***x*** *, t, d_i_)* is the fraction of cells killed from radiation since
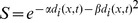
(5)represents the fraction of cells surviving from a dose d and fraction i from the linear-quadratic (LQ) radiation dose-response model [12]. The LQ model relates the dose in units (Gy) delivered to a region of tissue to a survival probability with parameters α (Gy−1) and β (Gy−2). Consistent with [7], we assume α/β = 10 Gy for early responding tumor tissue and 3 Gy for late responding tissues (normal brain) [12].

The PIRT Model assumes that glioma growth and invasion can be described by a diffusion process with coefficient D and proliferation rate ρ, where ρ is understood to represent the net change in population from cell reproduction and death. In the absence of the therapy response term, equation 2 is known to have a traveling wave solution that approaches a constant velocity, 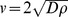, in agreement with observed linear radial growth in both low-grade and high-grade gliomas [13], [14].

**Figure 1:**
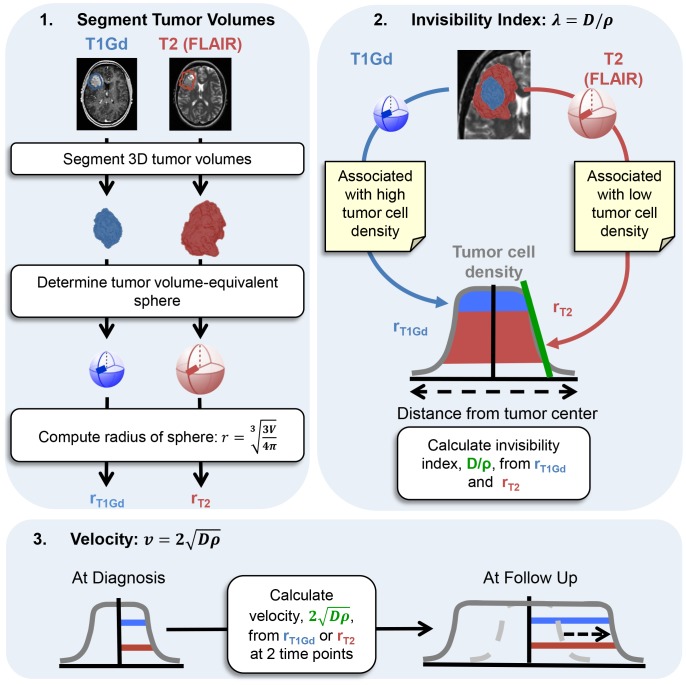
Parameter generation for the patient-specific biomathematical model. **1.** Determine radial measurements from serial T1Gd and T2/FLAIR magnetic resonance imaging. **2.** Compute the invisibility index (D/*ρ*) from intra-study T1Gd and T2/FLAIR radial measurements. **3.** Compute the radial velocity (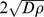) from serial T1Gd or T2/FLAIR radial measurements.

Following [13], [14], [7] and [15], to parameterize the model, we associate the enhancing regions on post-contrast T1-weighted (T1Gd) and T2-weighted (T2) or FLAIR MR imaging with surfaces of 0.80 k_t_ and 0.16 k_t_ tumor cell isodensity, respectively. While clinical target volumes determined from T2 and FLAIR imaging can differ in practice [16], both modalities are used interchangeably in this study, subject to their availability. Any differences in the segmented volumes would result in slightly larger effects than what is observed for intra-modality measurement uncertainty. We can compute the velocity of radial growth (*ν*) by evaluating serial imaging for a single MR modality, and by taking into account both T1Gd and T2/FLAIR imaging on a single day we can infer the gradient of tumor cell density peripheral to the imaging abnormality, represented by the ratio of diffusion to net proliferation (D/ρ). This ratio is a patient-specific “invisibility index” that characterizes subclinical disease burden relative to the imaging abnormality and results in a “tip of the iceberg” view of clinical imaging where tumors with larger proportions of cells below the imaging threshold, will have a larger invisibility index, and are considered more “diffuse”. Those with a higher percentage of tumor cells above the imaging threshold will have a small invisibility index and are considered to be relatively “nodular”. This characterization has prognostic implications for survival and response to radiotherapy [13] and is fundamental to generating patient-specific, biologically-optimized radiotherapy plans. Figure 1 illustrates the process of computing the net rates of proliferation (*ρ*) and diffusion (D) from routinely available MRI and a more detailed discussion of this model can be found in [13], [14], [7] and [15].

Normal brain tissue is modeled as a fixed volume not subject to radiation-induced cell killing or repopulation, and is computed at the time that radiotherapy begins. Normal tissue cell density (n) is defined to be the inverse of the tumor cell density (c) subject to the ratio of normal tissue carrying capacity to the tumor tissue carrying capacity (N_K_ = k_n_/k_t_) so that n = N_K_(1−c). The maximum normal tissue carrying capacity, k_n_ = 1.4×10^5^ cells/mm^3^, is computed by assuming an average of 2×10^11^ cells in a 1.4×10^6^ mm^3^ brain [11].

### Multi-Objective Evolutionary Algorithm (MOEA) for IMRT Optimization

We integrated the MOEA, as in [8], to generate deliverable IMRT plans that meet defined restrictions and are Pareto-optimal with respect to all decision criteria, i.e. plans for which any change to improve performance for one decision criteria will decrease performance for another [8]. Unlike the work presented in Holdsworth et al (2012) however [8], we applied a different set of decision criteria and optimization restrictions to generate more clinically acceptable dose distributions for a cohort of patients. Specifically, we provided the following two decision criteria to represent relevant clinical goals:

1. Minimize EUD to normal brain tissue.
2. Minimize the number of viable tumor cells after seven days of PIRT model simulation.

From the set of pareto-optimal plans, we chose the plan that results from giving equal importance to both decision criteria. More details of the MOEA can be found in [8].

### Optimization Restrictions

In addition to the decision criteria, we included several restrictions, summarized in table 1, to generate dose fractions that are comparable to the standard-of-care. Outside of the T2/FLAIR region, the total daily dose was limited to a peak of 2.5 Gy since the normal tissue toxicity for daily fractions that exceed this dose is unpredictable [17]. We also required that the per fraction EUD to normal tissue outside of the T1Gd enhancing region be less than or equal to the standard-of-care, ensuring that the total dose remains well below the TD 5/5 tolerance dose; the dose resulting in a 5% probability of complication within five years [18]. Within the T2/FLAIR volume, each dose fraction was limited to a peak of 5 Gy resulting in total doses of over 100 Gy to the center of target. In practice, these dose restrictions could be modified at the discretion of the clinician.

**Table 1:** Optimization Restrictions.

Maximum	Fraction/Dose	Region
5 Gy Peak	Fraction	Inside T2/FLAIR
2.5 Gy Peak	Fraction	Outside T2/FLAIR
65 Gy Peak	Dose	Outside T2/FLAIR
0.4–1.4 Gy EUD*	Fraction	Outside T1Gd

* Patient-specific.

### Patients

Six males and six females with a mean and median age of 57 and 60 years respectively, at the time of histologically diagnosed GBM (WHO grade IV) [19], consented to this study with approval by the local institutional review boards. This study includes the cohort studied in [7] and 3 additional patients, bringing the total number to 12, all of whom received radiation therapy and had at least two pre-treatment and one post-radiotherapy pair of T1Gd and T2/FLAIR MRI observations. All patient-specific model parameters were taken directly from or computed as described in [7] (table 2). For consistency, we chose to use the α determined from the post-treatment imaging for this study instead of using the relationship established in [7] to compute α from ρ using pre-treatment data. For a given patient, one plan will be better than another for all non-zero, reasonable values of α, since the same α is used when simulating for both the standard-of-care and the optimized plan and these plans are compared using EUD. Tumor volume increased following radiotherapy for patient 4, demonstrating no quantifiable benefit from radiotherapy and resulting in a negligible radiosensitivity: α = 0. From a modeling perspective, this corresponds to a situation in which the tumor growth rate is sufficiently large to overwhelm the response from radiotherapy resulting in positive net growth. This case presents a challenge to the ideas of BED and EUD and for that reason, we excluded this patient from this analysis, leaving 11 patients as the focus of our analysis.

**Table 2:** Patient Data.

Patient	Age	Sex	EOR*	Velocity(mm/year)	Net dispersalrate D (mm^2^/year)	Net proliferation rate ρ (/year)	Invisibility Index D/ρ (mm^2^)	Linear Quadratic radiosensitivity α (/Gy)	Pre-radiotherapyT2 Radius (cm)	Survival from firstMRI observation(months)	Concurrent chemotherapy during radiotherapy
1†	63	M	BX‡	50.9	18.43	35.13	0.52	0.162	3.15	88.0	TMZ¶
2	43	M	STR#	195.4	7.52	12.68	0.59	0.137	1.21	28.4	TMZ
3	53	M	BX	19.9	27.70	3.59	7.72	0.005	2.90	34.3	–
4	63	F	BX	21.9	7.88	15.24	0.52	0.000	1.15	13.2	TMZ
5	49	M	STR	42.3	8.90	50.29	0.18	0.265	2.01	18.8	BCNU||+TMZ
6	73	M	BX	24.3	10.82	13.68	0.79	0.076	1.97	71.9	TMZ
7	56	F	BX	53.1	50.71	13.88	3.65	0.028	2.00	12.8	TMZ
8	63	M	BX	20.1	12.64	7.99	1.58	0.032	2.04	15.6	TMZ
9	45	F	BX	21.1	6.56	17.04	0.38	0.084	1.92	21.3	TMZ
10§	30	F	BX	89.17	54.23	36.65	1.48	0.130	2.75	8.0	–
11§	73	F	BX	57.37	18.86	43.63	0.43	0.168	3.24	17.3	–
12§	68	F	BX	12.23	1.93	19.34	0.10	0.025	2.29	4.1	TMZ

* Extent of Resection.

† Still alive.

‡ Biopsy only.

¶ Temozolomide.

# Subtotal resection.

|| Carmustine.

§ Not included in Rockne 2010.

### Standard-of-Care Doses

Unless otherwise stated in the patients’ radiology reports, the standard-of-care plans were approximated based on the Stupp protocol [1], [7] with patients received an initial dose of 54 Gy in 30 fractions to the pre-treatment T2/FLAIR enhancing abnormality with a 2.5 cm margin, immediately followed by a boost dose of 9 Gy in 5 fractions defined by the pre-treatment T1Gd enhancing abnormality plus a 2 cm margin. Treatment was applied in equal fractions of 1.8 Gy on weekdays only and dose contours are fixed for the duration of treatment. The dose to the target included random variation of up to 5% of the prescribed dose to allow for patient setup errors. We approximated beam attenuation by assuming that the dose outside of the target contour decreases in proportion to the (radius at target edge)^2^/(radius outside of target)^2^ plus 3.5%/cm consistent with 6 MV x-ray fluence in tissue.

### Simulation of Optimized IMRT

Similar to [8], simulations incorporated MOEA-optimized dose fractions for each week of treatment by passing patient-specific PIRT model parameters and tumor cell density as inputs to the MOEA which returns a daily dose as described above. Tumor growth and response to this dose fraction was simulated for the next five days of treatment followed by two untreated weekend days. This process was repeated until the course of treatment ended. To ensure comparability to the simulated standard-of-care doses, optimized doses were converted to spherical symmetry before assessing the decision criteria and simulation. Simulations were run in accordance with the methodology presented in [7], except MATLAB’s *pdepe* function was replaced by in-house implementation of Crank-Nicolson numerical scheme [20]. Space is discretized in spherical symmetry where each spatial location *x_i_* in x indicates a point from the center of the tumor along a radius out to a maximum of 8 cm in intervals of.08 mm.

### Prognostic and Novel Metrics of Response

**Figure 2:**
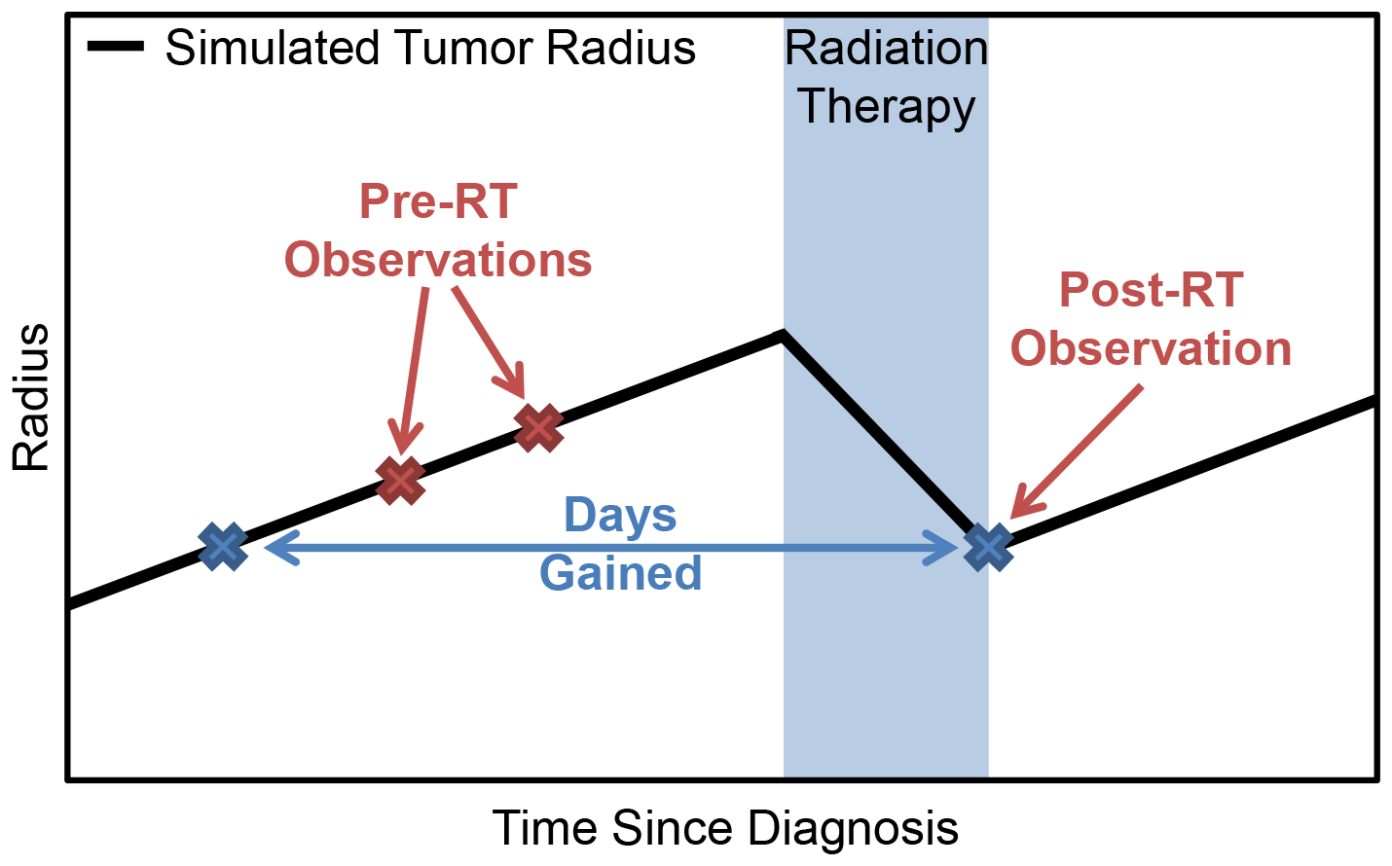
Computation of the Days Gained metric. The Days Gained score is the number of days between the post-radiation observation time-point and the time-point on the model-predicted, untreated growth trajectory where the tumor size is equal to the post-treat tumor size. The observations may be actual MRI observations or a chosen simulated time-point.

A novel metric of response called Days Gained (DG) was computed for each patient [15], [21]. The DG score for each patient is the number of days between the post-radiation time-point and the time-point on the model-predicted, untreated growth trajectory where the tumor size is equal to the post-treat tumor size, as illustrated in figure 2. This metric of response takes into account both patient-specific tumor growth kinetics and the direct effects of radiotherapy, and larger DG scores have been shown to be correlated with a progression-free and overall survival advantage [15], [21]. Other metrics, such as tumor control probability are not correlated with survival and ignore patient-specific relative rates of response [22].

### Parameter Sensitivity Analysis

We have estimated our inter-observer variability in tumor volume delineation to be 0.5 mm in spherically equivalent radius. Using this uncertainty in our data we computed the minimum and maximum D and D/ρ values possible given the measurement error and ran additional simulations, for each patient with these values. We also varied the ratio of LQ parameters α/β by +/− 20% to assess the sensitivity of our results to variations in the radiobiological parameters. The variation in input parameters as well as the impact on the results is illustrated in the form of error bars where appropriate in the results.

## Results

**Figure 3:**
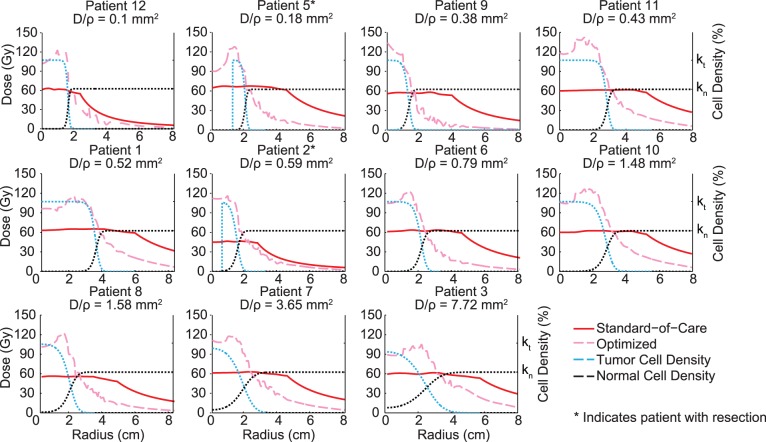
Simulated tumor and normal cell densities with clinical and optimized total dose. Patients are ordered according to tumor diffusivity, from least to greatest and the cell densities are taken at the pre-treatment timepoint. Dose in units Gray is on the left axis while cell density relative to the tumor cell carrying capacity is on the right side. The spatial distribution of the optimized plans is determined primarily by the patient-specific invisibility index (*D/ρ*), as patients with more nodular tumors (low *D/ρ*, e.g. Patient 12) receive more peaked optimized doses while those for patients with more diffuse tumors (high *D/ρ*, e.g. Patient 3) are more spread out along the invasive gradient of the outer edge of the tumor. Patients 2 and 5 show a cell density of zero in the center of the tumor due to subtotal resections.

**Figure 4:**
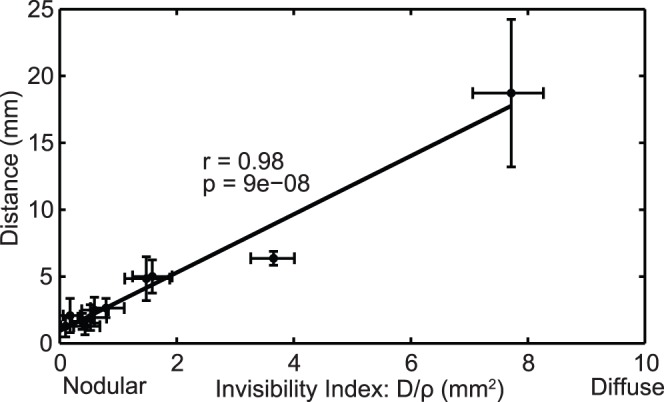
Spatial distribution of optimized plans versus the invisibility index. The radial distance between the 50% isodose radius and the 50% tumor cell isodensity radius versus the invisibility index (*D/ρ)* for the optimized plans. The horizontal error bars illustrate the range of patient-specific D/p values possible given the observed uncertainty in radial tumor measurements. The vertical error bars represent the minimum and maximum distance from simulations using the expected, minimum and maximum D/p values. The marker is plotted in the center of the range and the center values are positively correlated with Pearson’s correlation r = .98 with p-value = 9e-8, demonstrating that tumors with higher invisibility indices receive optimized doses with shallower gradients and larger high-dose volumes relative to tumor cell density.

**Figure 5:**
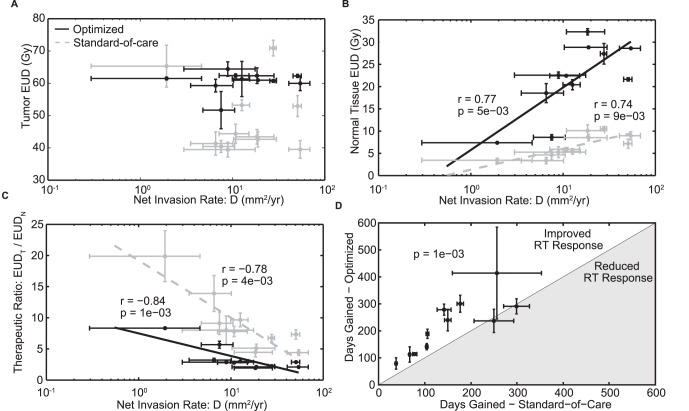
Metrics of treatment and response versus the diffusion coefficient, *D,* for both the standard-of-care (black) and optimized plans (grey) for all patients with corresponding linear regression lines and Pearson’s correlation coefficients and p-values where appropriate. **A.** Equivalent Uniform Dose (EUD) to tumor. 9 of 11 patients received lower tumor EUD, but there is no correlation with the PIRT model parameters. **B.** Equivalent Uniform Dose (EUD) to normal brain tissue outside of the T1Gd enhancing region. Although smaller in the optimized plans, the normal tissue EUD for both plans is positively correlated with the diffusion coefficient D with Pearson’s correlations r = 0.742 and 0.765; p = 0.009 and 0.005, for the standard-of-care and optimized plans respectively. **C.** Therapeutic ratio: the ratio of the tumor EUD to that of normal tissue**.** Therapeutic ratio for both plans is negatively correlated with the diffusion coefficient D with Pearson’s correlations r = −0.78 and −0.84; p = 0.004 and 0.001, for the standard-of-care and optimized plans respectively. **D.** Days Gained, the model-predicted, untreated growth time between first post-radiation tumor size and that predicted by the model prior to treatment, for both the standard-of-care and optimized plans for all patients.

The optimized plans deliver the maximum dose to the proliferating rim of the tumor, less dose to the center of the tumor and a decreasing gradient of dose on the periphery (figure 3). The spatial distribution of the optimized plans is determined primarily by the patient-specific invisibility index (D/ρ), as patients with more nodular tumors (low D/*ρ* and thus a steeper gradient of cell invasion) receive more peaked optimized doses while those for patients with more diffuse tumors (high D/*ρ* and thus a shallower gradient of cell invasion) are more spread out along the invasive gradient of the outer edge of the tumor (figure 3). This is further illustrated by looking at the radial distance between the 50% isodose radius and the 50% tumor cell isodensity radius, which is positively correlated with D/*ρ* for the optimized plans (Pearson’s correlation r = 0.98 and p = 9e-8) (figure 4), diffuse tumors (higher D/*ρ)* receive optimized doses with shallower gradients and larger high-dose volumes relative to tumor cell density. Parameter variation (visualized as the uncertainty bars around each data point in Figure 4 and 5) had a minimal effect on the dose shape with an average shift in 50% isodose radius of 3.2 mm and a maximum of 11 mm.

**Figure 6:**
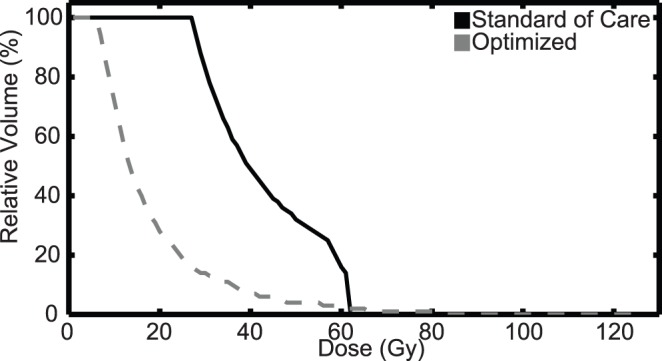
Dose-volume histogram showing the total dose delivered to normal brain tissue outside of the T1Gd abnormality for optimized and standard-of-care plans for patient 10. The lines intersect at 62% of the normal tissue volume, demonstrating that only 2% of the normal tissue receives a higher dose from the optimized plan.

In 9 of 11 patients, the EUD to tumor decreased significantly (2%–41%), but is not correlated with the degree of diffuse invasion (D) specific that patient (Figure 5a). When compared to the standard-of-care, the optimized doses resulted in a 67% to 93% decrease in normal tissue EUD outside of the T1Gd enhancing region (figure 5B). Both the standard-of-care and optimized doses result in normal tissue EUD that are positively correlated with the net rate of invasion *D* (Pearson’s correlation = 0.742, 0.765, p = 0.009, 0.005, respectively) (figure 5B). An average variation of 500 cGy was observed to result from parameter uncertainty (bars in Figure 5A and B). This large decrease in normal tissue EUD drives an increase in therapeutic ratio for all patients with increases ranging from 50% to 265% (figure 5C). As in [15], [21], we estimated the treatment effect of the standard-of-care and optimized plans in terms of our Days Gained response metric and found a measurable improvement in 9 of 11 patients with a statistically significant improvement in the mean Days Gained from optimized therapy (p = .0014, Figure 5D). The reduction in dose to normal tissue is emphasized further when looking at a dose-volume histogram comparing an optimized dose to the standard-of-care for representative patient 10 (Figure 6). Only 2% of normal tissue volume outside of the T1Gd abnormality receives a higher dose than the standard-of-care for this patient.

## Discussion

Despite tremendous effort to improve the prognosis of patients with glioblastoma over the past five decades, very little progress has been made to improve median survival. To this end, we have focused our research efforts to understanding the underlying kinetics of disease progression and have developed a means to improve upon the standard-of-care radiation protocol using a mathematical model and algorithm for radiotherapy optimization. The current one-size-fits-all approach in radiation treatment of glioblastoma ignores patient-specific tumor kinetics by adding standard margins in an attempt to capture subclinical disease, resulting in significant volumes of normal brain tissue receiving large doses and limiting the dose delivered to the tumor.

By utilizing a patient-specific approach for radiotherapy plan generation, we found that the relative diffuse extent of each tumor, expressed as the ratio of PIRT model parameters, *D/ρ*, drives the non-uniform spatial distribution of the optimized plans (Figure 4). For example, in Figure 3, nodular patient 6 and diffuse patient 7 appear similar on T2/FLAIR MRI leading to almost identical clinical standard-of-care treatment volumes (red curve), but they have very different rates of cell diffusion (D = 10.82 and 50.71 mm^2^/yr, respectively) and invisibility indices (*D/ρ* = 0.79 and 3.65 mm^2^, respectively). The peak of the optimized doses for each patient is between 100 and 130 Gy, about twice the maximum dose for the standard-of-care, so the optimized 50% isodose region corresponds roughly to the standard-of-care high-dose region. While the peak dose is high relative to the standard-of-care, it is not necessarily prohibitive in the context of previous dose escalation studies that have shown no increase in normal brain toxicity with peak doses up to 90 Gy [23]. The use of such high doses has traditionally been avoided due to increased risk of radiation necrosis, but the selection of such doses in the optimization process may indicate one of the reasons for the poor control of gliomas. It also highlights the need for better models of brain response to radiation so that the decision model can better balance these competing goals.

As illustrated in figure 4, the radius of the high-dose region for each optimized plan relative to the 50% tumor cell density radius is much larger for the high *D/ρ* patient and grows linearly relative to *D/ρ* across the entire cohort. This correlation held when accounting for tumor radius measurement error of 0.5 mm and a 20% variation in the ratio of LQ model parameters α/β (figure 4). For all patients, the high-dose region encompasses the 50% tumor cell density radius and therefore the majority of the tumor, including the most proliferative and radiosensitive regions. In the center of the tumor, where the cell density approaches the carrying capacity of the tissue and little to no model-predicted radiation effect and proliferation occur, typically due to hypoxia or necrosis, the optimized doses deliver less than the maximum dose. The optimization places little importance to this area since there is no relative benefit in terms of the decision criteria. Since the algorithm is dose-limited by regions of normal tissue, the optimization focuses on delivering the highest dose to the proliferating rim of the tumor, where it has the largest effect (figure 3).

The combination of restrictions and decision criteria provided to the MOEA resulted in optimized plans that reach high peaks inside the T1Gd enhancing region but are much lower in the surrounding normal brain tissue than the standard-of-care. In areas where the model predicts little to no normal tissue, the optimization algorithm will seek the maximum possible dose that can be delivered given any local and distant restrictions. Very steep dose gradients are deliverable using IMRT, so the dose remains very low outside of the center of the tumor while the dose in the center is escalated, avoiding extended low-dose regions that are common when using IMRT to treat standard targets with large margins. The narrower margins result in significantly reduced normal tissue and tumor EUD (9 of 11 patients) and increased therapeutic ratio (figure 5C). The combination of high peak doses and lower EUD to tumor is the result of the tumor cell density decreasing much slower than the volume increases further away from the center of the tumor. Since EUD is a volume-weighted metric, this low-density, high-volume region is a non-trivial contributor to tumor EUD and results in lower tumor EUD despite high peak doses. The Days Gained scores for all but 2 patients improve, suggesting that the optimized plans succeed in delivering a much more effective spatial distribution of dose, even with reduced EUD to tumor.

The location of the high-dose region is appropriate since dose escalation studies have demonstrated that approximately 90% of failures occur “in-field”, defined as having at least 80% of the recurrence volume inside the high dose region [23], [24]. There is a small region of mostly normal tissue (between the T1Gd and T2/FLAIR boundaries) that receives a very large dose (<2% above 60 Gy), and these areas should be considered when designing the treatment volumes to avoid eloquent areas. From a dose escalation perspective, the optimized plans are an improvement over those used in clinical studies that include whole-brain irradiation or non-patient-specific dose distributions [23], [25].

The integrated model takes advantage of patient-specific growth kinetics, metrics of response at multiple points in time and an adaptive optimization process to generate plans that deliver dose to tumor much more efficiently than the standard-of-care and current state-of-the-art “dose-painting” approaches. While the focus in this manuscript is a comparison with the current standard-of-care, the optimized doses presented here could be compared to other experimental dosing strategies that also take advantage of PIRT model-predicted tumor cell density to determine target boundaries.

## Conclusions

The diffusely infiltrative nature of glioma presents a challenge to dose optimization as the benefit of additional dose to the target is balanced by the cost of increased toxicity to the normal tissue. This work highlights the potential of using a mathematical model and a multiobjective evolutionary algorithm for intensity-modulated radiation therapy optimization to generate individualized radiation treatment plans. Simulations of the PIRT model using a MOEA for IMRT optimization generated patient-specific, biologically-guided plans that outperformed the standard-of-care in silico by delivering lower normal tissue EUD, improved therapeutic ratios and higher Days Gained scores. The strong relationship between the diffusion coefficient D and both normal tissue EUD and therapeutic ratio (figure 5) identifies patients with a higher risk of normal tissue complications who may be targets for alternative treatment strategies.

While this model is promising, it relies on many simplifying assumptions such as instantaneous, radiation-induced cell death, a radio-response parameter that may include the effects of concurrent chemotherapy, radiation necrosis and pseudoprogression. In addition, the patient population to which this model can be applied is limited to patients without extensive surgery due to the difficulty in modeling resection in spherical symmetry. Additional parameters can be added to account for the above and this analysis can be undertaken in a three-dimensional, anatomically correct brain to model larger resections, incorporate grey and white matter distributions, actual tumor location and dose to anatomical structures as typically addressed in clinical treatment planning. The optimized plans can then be compared directly to the clinical plans and adjusted accordingly. The decision criteria can also be improved to include spatially-defined biological endpoints, dose to specific structures or any other quantifiable, clinically relevant metrics. With these further improvements, we believe this method has great potential to provide insight into and improve the standard-of-care radiation therapy protocol.
